# Radar Compound Jamming Recognition Based on Image Segmentation and Fused Attention Residual Network

**DOI:** 10.3390/s25072124

**Published:** 2025-03-27

**Authors:** Peishan Li, Jian Yang, Jiaao Lin

**Affiliations:** China Academy of Launch Vehicle Technology, No. 165, South 4th Ring East Road, Fengtai District, Beijing 100076, China; summermax@163.com (J.Y.); 18811691131@163.com (J.L.)

**Keywords:** compound jamming, jamming recognition, residual network, attention mechanism

## Abstract

With the increasing complexity of modern electromagnetic environments, radar systems are not only affected by single jamming signals but also by compound jamming, which consists of additive combinations of multiple jamming types. Compound jamming is difficult to recognize due to a wide array of diverse compound patterns. To address this issue, this study proposes a method for the segmentation and recognition of compound jamming signals. First, a jamming segmentation module based on image segmentation techniques is designed to segment the compound jamming in the time–frequency domain, which is obtained by short-time Fourier transform (STFT). Subsequently, an enhanced residual network (ResNet) incorporating a spatial-channel fused attention mechanism (SCFAM) is proposed to effectively capture multi-level features and recognize the segmented jamming signals. The experimental results demonstrate that the proposed method achieves a recognition accuracy of 98.60% for compound jamming, outperforming three classical approaches. Additionally, this method exhibits superior performance in recognizing untrained types of compound jamming, highlighting its robustness and generalization capability.

## 1. Introduction

Radar, an active sensor system for environmental perception and target detection, utilizes electromagnetic waves to detect targets and is crucial in fields like intelligent transportation, environmental monitoring, and maritime navigation. However, the development of Digital Radio Frequency Memory (DRFM) technology has introduced unprecedented jamming challenges to radar systems [1]. DRFM-equipped jammers can quickly replicate, modulate, and retransmit radar signals, generating complex jamming signals. In complex electromagnetic environments, compound jamming strategies that combine multiple types of jamming signals are frequently employed. These signals degrade the quality of target echo signals, making it difficult for radar systems to distinguish between genuine targets and jamming signals [2,3].

To mitigate these threats and improve radar reliability, anti-jamming technologies have emerged. As the initial and pivotal step in radar anti-jamming, jamming recognition is of fundamental significance. Only by accurately identifying the type of the jamming can a reliable foundation be laid for the formulation of subsequent anti-jamming strategies. Different types of jamming signals exhibit distinct characteristics. For example, noise jamming typically appears as random electromagnetic noise, while deception jamming mimics the features of target echo signals to mislead radar systems. Compound jamming has become a major research hotspot due to its diverse combination patterns and complex characteristics, which are difficult to accurately recognize [4].

Recognition methods for jamming mainly include classifier algorithms based on feature dataset and intelligent algorithms based on deep learning [5,6,7]. For the feature dataset, common classifiers such as random forest, gradient boosting, and support vector machines are used to recognize jamming [8,9]. With the development of deep learning technology, many researchers have combined convolutional neural networks (CNNs) with jamming recognition [10]. A backbone composed of convolutional blocks is proposed for learning advanced features and simultaneously recognizing jamming signals [11]. For open set, references [12,13] propose an open world recognition method based on residual convolutional autoencoders and two models for radar jamming open set recognition, respectively, to implement the classification of known and unknown patterns. Reference [14] proposes a weakly supervised transformer to address the issues of performance degradation under weak supervision, which is of greater practical value for enhancing the anti-jamming capabilities of radar. A residual convolutional neural network with a convolutional block attention module for a radar deception jamming recognition algorithm in the presence of extended targets is proposed in reference [15].

These above-mentioned references all focus on the identification of various single-type jamming. In addition, some scholars have also conducted relevant research on the identification of compound jamming for radar. For compound jamming signals consisting of suppression and deception jamming, reference [16] puts forward a recognition approach relying on a dual-channel neural network along with feature fusion. The suggested fusion subnetwork can enhance the recognition performance. Reference [17] focuses on compound suppression jamming signals. It proposes a novel jamming recognition network based on robust power spectrum features, which is used to identify ten types of suppression jamming signals, including four single jamming and six compound jamming signals. However, in the studies above, compound jamming was regarded as a new type of jamming for recognition. In this case, the number of jamming types was the combination number of jamming types, which was large and difficult to completely train. Reference [18] put forward a “Segmentation + Recognition” strategy to overcome the label limitation of the supervised learning method in the recognition process. Furthermore, it developed a compound jamming recognition method based on source segmentation. However, this method assumes that each channel in the radar multi-channel receiving model receives only one jamming type. Thus, when the jammer sends composite jamming, the method fails, indicating the need for better compound jamming recognition techniques in a modern radar’s complex electromagnetic environment.

To solve these problems, we plot a time–frequency diagram of compound jamming using the short-time Fourier transform (STFT) for better separability. Then, a compound jamming segmentation module based on the image filter is designed to obtain the segmented time–frequency diagram. An augmented residual network (ResNet) with a fused attention mechanism is constructed to achieve the recognition of the segmented jamming. The proposed algorithm can recognize new compound jamming patterns rather than specific compound jamming patterns that are trained by the network. The main contributions of this study are as follows:

1. A compound jamming segmentation module based on Gabor filtering and k-means clustering is proposed to segment the time–frequency diagram of compound jamming;
2. ResNet is optimized using the spatial-channel fused attention mechanism (SCFAM) on a hierarchical scale to extract critical jamming features for recognition;
3. The experimental results show that the proposed algorithm has better recognition performance on the validation dataset and can recognize untrained patterns of compound jamming in the test dataset.

The content of this paper is structured as follows. In Section 2, we delve into the mechanisms of six typical jamming types and additional compound jamming. Section 3 details the approach of image segmentation and the enhanced residual network integrated with a fused attention mechanism. In Section 4, we present the relevant simulation results and their corresponding analyses. Finally, Section 5 concludes this study by summarizing the key findings and contributions.

## 2. Radar Jamming Model and Preprocessing

### 2.1. Single Jamming Model

According to different jamming mechanisms, radar jamming can be divided into suppression jamming and deception jamming. To comprehensively validate the generalization capability of the proposed method, this study meticulously selects six representative jamming signals as the research subjects. Among them are three typical suppression jamming types, namely, radio frequency jamming (RF), frequency-modulation jamming (FM), and amplitude-modulation jamming (AM). Additionally, three typical deception jamming types are included, i.e., intermittent sampling and repeating jamming (ISRJ), slice reconstruction jamming (CI), and smeared spectrum jamming (SMSP).

RF jamming works by transmitting extremely powerful radio frequency signals, which generate noise within the radar’s operating frequency band. This noise directly overwhelms the radar echo signals. Thus, it becomes extremely difficult for the radar to detect the target. This kind of jamming represents the most fundamental and straightforward form of jamming.

(1) $$j_{RF}\left(t\right)=U_{n}\left(t\right)\cos\left(\omega_{j}t+\phi \right)$$

where envelope $U_{n}\left(t\right)$ follows a Rayleigh distribution, the phase ϕ satisfies a uniform distribution in the interval $\left[0,2\pi \right]$ and is independent of $U_{n}\left(t\right)$, and the carrier frequency $\omega_{j}$ is a constant.

FM jamming is based on the principle of signal frequency alteration. By modulating the frequency of the interference signal, it disrupts the frequency characteristics of the radar signal, causing errors in the radar’s frequency calculation and target information extraction. This represents a category of interference methods that exploit the frequency-modulation characteristics.

(2) $$j_{FM}(t)=U_{j}\cos\left[\omega_{j}t+2\pi K_{FM}\int_{0}^{t}u(t^{\prime })dt^{\prime }+\phi \right]$$

where the modulation noise $u(t^{\prime })$ is the zero-mean Gaussian white noise, $U_{j}$ is the amplitude of the noise-frequency-modulated signal, $\omega_{j}$ is the central frequency of the noise-frequency-modulated signal, and $K_{FM}$ is the frequency-modulation slope.

AM jamming mainly changes the amplitude of the signal. By leveraging the principle of amplitude modulation, it distorts the amplitude of the radar-received signal, affecting the radar’s judgment of the target echo intensity and the measurement of the target distance. It is a typical example of interference based on the amplitude-modulation principle.

(3) $$j_{AM}(t)=\left(U_{0}\left(t\right)+U_{n}\left(t\right)\right)\cos\left(\omega_{j}t+\phi \right)$$

where $U_{0}\;$is the amplitude of the carrier voltage, and $\omega_{j}$ is the carrier frequency. The modulation noise $U_{n}\left(t\right)$ is the Gaussian white noise with a mean of zero and a variance of ${\sigma_{n}}^{2}$, and it is a stationary random process.

ISRJ jamming takes advantage of the intermittent sampling and retransmission of radar signals. It skillfully inserts false sampling signals into the received radar signals, creating false targets and interfering with the radar’s target detection and tracking. It is a representative of deceptive jamming based on the principles of signal sampling and retransmission.

(4) $$j_{ISRJ}(t)=\sum_{n=0}^{N-1}\mathrm{rect}\left[\frac{t-\frac{\tau }{2}-\left(n-1\right)T_{s}}{T}\right]s\left(t\right)$$

where N represents the number of samplings, T denotes the pulse width, $T_{s}$ is the period of intermittent sampling, t stands for the sampling time of intermittent sampling, and $s\left(t\right)$ is the radar-transmitted signal.

CI jamming is like ISRJ as it can also generate false targets, making it difficult for the radar to correctly identify and process signals. However, CI distinguishes itself from ISRJ as, in addition to false-target generation, it can manipulate the characteristics and trajectories of the targets.

(5) $$j_{CI}\left(t\right)=\sum_{l=0}^{N-1}p\left(t-\frac{lT_{p}}{MN}\right)$$

where M represents the number of slices, and N denotes the number of time slots in each slice. Thus, the original signal is segmented into M×N segments. The time length of each slice is expressed as $\frac{T_{p}}{M}$, and the signal time width of each time slot is $\frac{T_{p}}{\left(M\cdot N\right)}$.

SMSP jamming extends the spectrum of the jamming signal into the spectrum range of the radar signal, disrupting the spectral characteristics of the radar signal. It is a typical example of interference that exploits spectral characteristics.

(6) $$j_{SMSP}(t)=\sum_{l=0}^{N-1}j_{0}\left(t-\frac{nT_{p}}{N}\right)$$

where $J_{0}\;$is the interfering sub-pulse, and it is expressed as follows:

(7) $$j_{0}(t)=A_{j}exp\left(j2\pi \left(f_{c}+\frac{1}{2}k^{\prime }t^{2}\right)\right)$$

where $A_{j}$ is the amplitude of the interference, $f_{c}$ is the central frequency, and $k^{\prime }=Nk$ represents the frequency-modulation slope that compresses the signal. By altering k and the number of replications N, the jamming mode of spectrum dispersion jamming can be changed.

### 2.2. Compound Jamming Model

In the context of modern radar systems, the coupling of different types of jamming poses a formidable challenge. The compound modes of jamming include three types: multiple-suppression jamming compound, multiple-deception jamming compound, and the compound jamming of suppression and deception. Suppression jamming mechanistically submerges signal by noise; thus, it is difficult to segment multiple-suppression compound jamming. Furthermore, high-power jammers are required to achieve suppression jamming, and multiple-suppression compound jamming can result in additional economic burdens. Considering economic and application factors, we only take into account additive compound jamming signals [14]. Compound jamming is obtained by adding N kinds of jamming.

(8) $$j_{com}\left(t\right)=\sum_{i=1}^{N}j_{i}\left(t\right)$$

where $j_{com}\left(t\right)$ is the compound jamming signal in time domain, and $j_{i}\left(t\right)$ is the ith single jamming signal. The Fourier transform of $j_{com}\left(t\right)$ is $J_{com}\left(\omega \right)$.

(9) $$J_{com}\left(\omega \right)=\sum_{i=1}^{N}J_{i}\left(\omega \right)=\sum_{i=1}^{N}\int_{-\infty }^{\infty }j_{i}\left(t\right)e^{-i\omega t}dt$$

where $J_{com}\left(\omega \right)$ is also obtained by adding N single jamming signals in the frequency domain, and $\int_{-\infty }^{\infty }j_{i}\left(t\right)e^{-j\omega t}dt$ is the Fourier transform of $j_{i}\left(t\right)$, where $i=\sqrt{-1}$ and ω is the angular frequency with a unit of radians per second. We can conclude from the above formula that compound jamming creates the aliasing problem in both the time domain and frequency domain.

When jammers implement suppression jamming and deception jamming at the same time, the generated pattern of compound jamming has both suppression and deception characteristics. Suppression jamming increases the difficulty of the radar in detecting the jammer, improves the safety of the jammer in implementing deception jamming, and can also make the radar incapable of distinguishing between true and false targets through false echo signals, thus effectively improving the jamming ability.

Taking the compound of RF and ISRJ as an example, the compound jamming signal is expressed as follows:

(10) $$J_{com}\left(t\right)=k_{1}U_{n}\left(t\right)\cos\left(\omega_{j}t+\phi \right)+k_{2}\sum_{n=0}^{N-1}rect\left[\frac{t-\frac{\tau }{2}-\left(n-1\right)T_{s}}{T}\right]s\left(t\right)$$

where $k_{1}$ and $k_{2}$ are compound coefficients with different compound intensities. In this study, we mainly consider the $k_{2}/k_{1}>2\;situation,inwhich$ suppression jamming cannot submerge deception jamming.

The deception effect of multiple-deception jamming compound on the radar can be further enhanced, becoming a deception jamming with more diverse false-target numbers, positions, and shapes, which can have a better jamming effect. Deception jamming focuses on generating false targets rather than high-power suppression signals; thus, we set the compound coefficients to be the same.

Compound jamming is difficult to recognize for various types and parameters through additive compound. Therefore, it is a crucial step to segment single jamming from compound jamming.

### 2.3. Time–Frequency Analysis

When different types of jamming are coupled, aliasing occurs in both the time domain and frequency domain, making it extremely difficult to segment and distinguish them. Time–frequency analysis, which encompasses both time and frequency information, is a method capable of revealing the local characteristics of signals.

**Figure 1:**
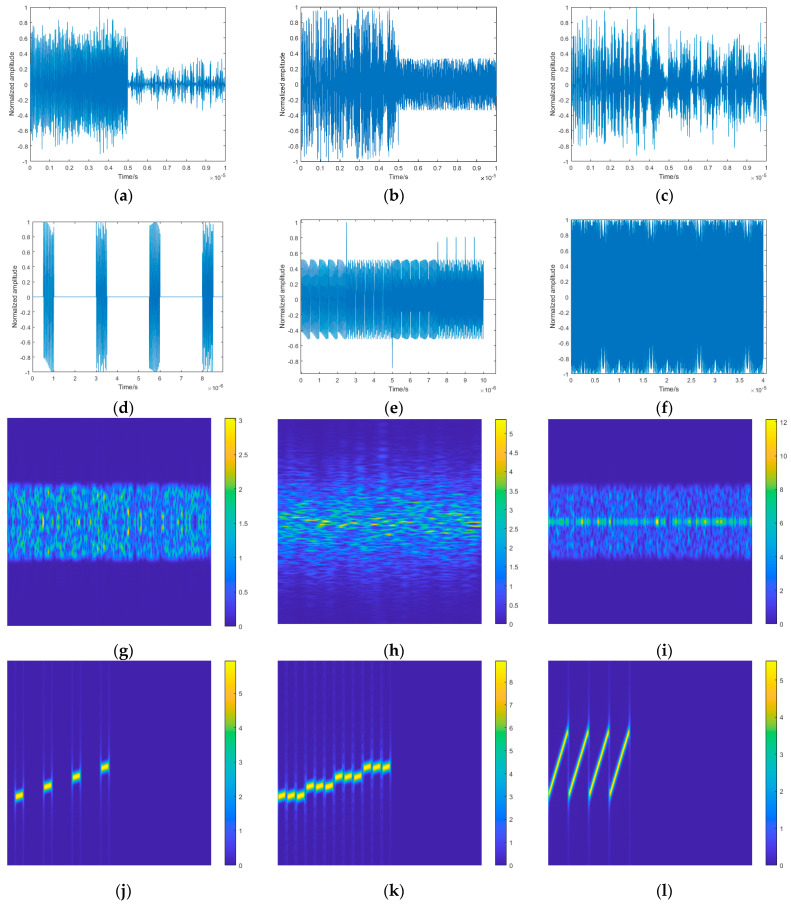
Time domain waveforms and time–frequency domain waveforms of six different jamming signals: (**a**–**f**) time domain waveforms for RF, FM, AM, ISRJ, CI, and SMSP, respectively; (**g**–**l**) time–frequency domain waveforms for RF, FM, AM, ISRJ, CI, and SMSP, respectively.

The short-time Fourier transform (STFT) is one of the most extensively and prevalently utilized time–frequency analysis methods in the field of signal processing. This process allows for a more accurate decomposition of the mixed signals, enabling the identification and analysis of individual jamming components.

(11) $${STFT}_{com}\left(t,f\right)=\int_{-\infty }^{+\infty }j_{com}\left(\tau \right)h\left(\tau -t\right)\exp(-j2\pi ft)d\tau$$

where $h\left(t\right)$ is the window function. The Hamming window, with a wide main lobe and significant side-lobe attenuation that reduces spectral leakage, is used for jamming signal time–frequency analysis in this study. The time–frequency domain waveforms of the six types of jamming studied in this study are shown in Figure 1. During the transformation, the frequency content of each short-time segment can be precisely determined. Thus, it enables the identification of jamming.

## 3. Proposed Approach

### 3.1. Compound Jamming Segmentation Module

**Figure 2:**
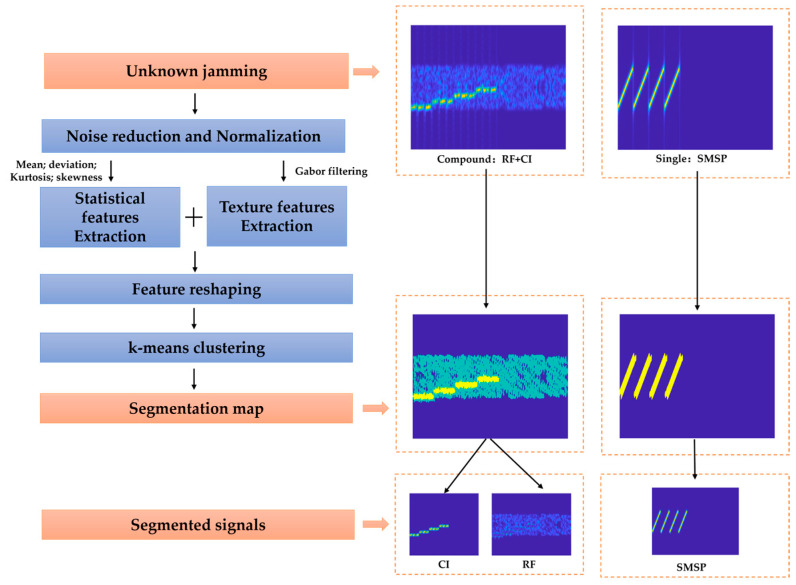
The flow chart of jamming segmentation module.

Given that diverse forms of jamming manifest unique statistical and textural properties within the time–frequency domain waveform, image segmentation techniques present a viable approach for the segmentation of compound jamming. This module employs the Gabor filtering and k-means clustering algorithms to accomplish the segregation of compound jamming signals in the time–frequency diagram, as shown in Figure 2.

The processing procedure is initiated with noise reduction and normalization steps. During this stage, the input time–frequency diagram of compound jamming is preprocessed. The jamming signal is rendered to satisfy the prerequisites for subsequent processing through a reduction in noise influence and the implementation of normalization operations.

Gabor filtering is implemented to extract two crucial categories of features from the signal: global statistical features and local texture features. The extraction of statistical features primarily centers on four statistical metrics of the signal, including the mean value, deviation, kurtosis, and skewness. In terms of the extraction of texture features, Gabor filters are resorted to for capturing the texture information within the time–frequency domain waveform of the signal, thereby furnishing a more multifaceted information dimension for the segmentation of jamming.

The scale and orientation parameters of the Gabor filter resemble those of the human visual system, making it highly suitable for texture representation and discrimination. The formula for a two-dimensional Gabor filter is as follows:

(12) $$g\left(x,y;\lambda ,\theta ,\varphi ,\sigma ,\gamma \right)=\exp\left(-\frac{{x^{\prime }}^{2}\gamma^{2}{y^{\prime }}^{2}}{2\sigma^{2}}\right)exp\left(i\left(2\pi \frac{x^{\prime }}{\lambda }+\varphi \right)\right)$$

where $x^{\prime }=xcos\theta +ysin\theta$, $y^{\prime }=-xsin\theta +ycos\theta$, λ is the wavelength, θ is the orientation, φ is the phase offset, σ is the standard deviation of the Gaussian window, and γ is the spatial aspect ratio.

After feature extraction, a reshaping operation is performed on the features obtained. This process arranges the features into a format conducive to cluster analysis. Promptly following this, the k-means clustering algorithm is employed. The objective of the k-means clustering algorithm is to partition n data points into k clusters, minimizing the sum of the squared distances from each data point to the centroid of its assigned cluster. The set of reshaped features is $X=\left\{x_{1},x_{2},\ldots ,x_{n}\;\right\},$ and the centers of cluster are $C=\left\{c_{1},c_{2},\ldots ,c_{k}\;\right\}$. The objective function is as follows:

(13) $$R=\sum_{i=1}^{n}\sum_{j=1}^{k}{\left\|x_{i}-c_{j}\right\|}^{2}$$

Within the code implementation, the k-means function is invoked to conduct clustering analysis on the feature vector. Consequently, the clustering labels and the centers of the clusters are successfully derived. Ultimately, a segmentation map is generated, which can clearly display the distribution of different jamming and provide strong support for subsequent jamming recognition.

In the right-hand side of Figure 2, the segmentation results for unknown jamming, denoted as RF + CI and SMSP, are exhibited. In the results of the segmentation map, the background region of the time–frequency diagram is color-coded in blue, whereas the time–frequency diagram corresponding to the two different interferences is marked in green and yellow. The results indicate that the compound jamming segmentation module accomplishes a proficient segmentation of the time–frequency diagram of the compound jamming. In the case of a single jamming signal input, the module simply outputs that single signal. This flexibility allows our method to be applicable across various jamming situations.

### 3.2. Residual Network with Fused Attention Mechanism

A 34-layer residual network is selected as the main part of the proposed network because of its strong capacity to solve the degradation problem and its versatility compared to traditional convolution networks. This residual network includes convolutional layers, four residual layers, an average pooling layer, and a fully connected layer. The detailed parameters are shown in Table 1. It also illustrates the allocation of the attention mechanism throughout all layers.

**Figure 3:**
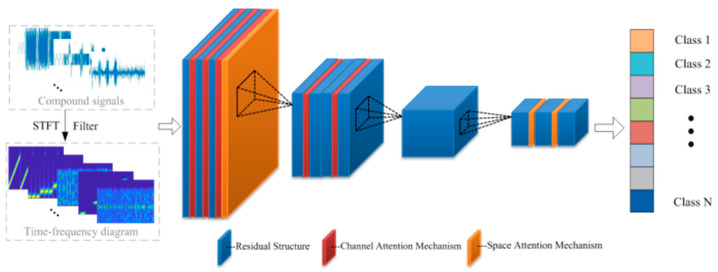
Structure of proposed residual network with fused attention mechanism.

The proposed recognition network contains three spatial attention modules and five channel attention modules, as depicted in Figure 3. The algorithm can focus on the key recognition parts through an attention mechanism. However, randomly adding the attention mechanism raises complexity and lowers accuracy. Therefore, it is necessary to design an attention mechanism suitable for jamming recognition.

In the front layers of the residual network, the shallow information of the input feature map is trained. Adopting the spatial attention mechanism (SAM) can make the network better locate the position information worthy of attention in the input diagram, facilitating the subsequent layers to extract more important feature information. Therefore, the SAM is adopted between the convolution layer and the first residual structure.

(14) $$S_{SAM}=\sigma \left(f^{7\times 7}\left(\left[A_{avg};A_{max}\right]\right)\right)$$

where $\sigma ,{\;A}_{avg},{\;and\;A}_{max}\;$denote the sigmoid function, the average pooled feature map, and the max pooled feature map, respectively, and $f^{7\times 7}$ represents a convolution operation with a 7×7 kernel. The output feature map O can be obtained with the formula $O=I\times S_{SAM}$, where I is the input feature map.

In the latter several layers of the network, the abstract feature is trained; thus, the channel attention mechanism (CAM) is a more suitable addition to learn which feature map of the 512 channels is more important. The CAM is added to the second and fourth residual structures.

(15) $$S_{CAM}=\sigma \left(M_{avg}+M_{max}\right)$$

where $M_{avg}$ and $M_{max}$ are two pooled vectors through shared multilayer perceptions (MLPs). The output feature map Y can be obtained by $Y=X⊗S_{CAM}$, where X is the input feature map and ⊗ represents element-wise multiplication.

Among them, in the last residual block of the first residual structure, a channel attention module is connected in series with the spatial attention module. The series connection of the spatial and channel attention mechanisms multiplies the channel attention feature map and the spatial attention feature map with the original input feature map element by element, achieving attention focusing in the channel and space dimensions.

Notably, the third residual structure is unmodified, which is the main body of the 34-layer residual network, with six residual blocks, and it is the main module for input image feature extraction. Adding an attention mechanism to this layer increases the risk of overfitting.

By adding the fused spatial-channel attention mechanism to the residual network, combining the advantages of spatial attention and channel attention, the algorithm can enhance the generalization performance of the recognition network and adaptively adjust the feature extraction strategy according to different input scenarios, different parameters, and different situations where there are a few incomplete steps in compound segmentation. The network can focus on features such as the shape and key texture of the object through the attention mechanism, thus generalizing better to various practical application scenarios.

### 3.3. Training Strategy and Optimization Algorithm

The attention module can help the algorithm focus on important features; however, due to the increase in the complexity of the residual network, the calculation time increases. Therefore, appropriate training strategies and optimization algorithms are required.

Data enhancement performs a series of transformations on the original jamming training dataset to increase its diversity so that the algorithm can learn more general features and reduce overfitting to specific data patterns. When generating each type of jamming training image dataset, a random area is selected from some of the original images for random cropping. If the size of the original image is H×W×C and the cropping is randomly selected within a certain range $x\in \left[0,H-h\right],y\in \left[0,W-w\right],$ where the feature of the image is not completely lost, the size of the cropping image is h×w×C. At the same time, by considering the center of the image $\left(c_{x},c_{y}\right)$ as the rotation center, a random angle θ is selected for rotation.

(16) $$x^{\prime }=\left(x-c_{x}\right)\cos\theta -\left(y-c_{y}\right)\sin\theta +c_{x}$$

(17) $$y^{\prime }=\left(x-c_{x}\right)\sin\theta -\left(y-c_{y}\right)\cos\theta +c_{y}$$

where $\left(x^{\prime },y^{\prime }\right)$ is the rotated coordinates of the image.

The early stopping strategy monitors the performance of the algorithm on the validation dataset during each training epoch. When the performance does not improve after a certain number of training rounds or begins to decline, training is stopped early. For the residual network integrated with an attention module, the early stopping strategy becomes especially vital. Attention mechanisms in neural networks, while enhancing the model’s ability to focus on relevant features, also introduce additional complexities and parameters. In such cases, the early stopping strategy acts as a safeguard. It ensures that the training process halts before the model reaches a state of overfitting, thereby preserving the model’s generalization ability. For the optimization algorithm, we use the adaptive moment estimation (Adam) during the network training process.

(18) $$\theta_{t}=\theta_{t-1}-\frac{\alpha }{\sqrt{{\hat{v}}_{t}}+\varepsilon }{\hat{m}}_{t}$$

where α is the learning rate, ε is a very small constant to prevent the denominator from being zero, and ${\hat{m}}_{t}$ and ${\hat{v}}_{t}$ are bias corrections for the first and second moment. Parameter θ updates from time t−1 to t.

## 4. Experiment and Analysis

### 4.1. Datasets

To evaluate the recognition performance of the ResNet-SCFAM proposed in this study, six classical single jamming and four compound jamming are simulated to train the algorithm, whose parameters are listed in Table 2. After being processed by the segmentation module, the four types of compound jamming signals are integrated into the six-category single jamming training datasets. This design strategy is aimed at augmenting the robustness of the ResNet-SCFAM when confronted with the time–frequency spectrograms of partially defective jamming signals that have been subjected to compound segmentation procedures. Notably, it considerably bolsters the accuracy of the network in differentiating the untrained composite jamming signals present in the test dataset.

The radar-transmitted signal is an LFM waveform, and jammers perform jamming operations based on the received waveform, The jamming-to-noise ratio (JNR) of the jamming signal is varied from −10 to 10 dB with two-step increases. To enhance the diversity of the dataset, random interpolation is performed on different parameters of different jamming within the parameter range to generate jamming signals and obtain different shapes of jamming time–frequency diagrams. The number of data points in each jamming type is 550, of which 100 are signals segmented from the compound jamming signal. The total data maps are divided into a training dataset and a validation dataset in a ratio of 8:2; thus, the total training dataset contains 2640 signals and the validation dataset contains 660 signals. To unify the size of the input matrix of the network, the input image size is scaled to 224 × 224 × 3, where 224 × 224 is the width and 224 pixels is the height, and 3 represents the three-channeled RGB of the image.

### 4.2. Experimental Settings

The recognition network designed in this study has a total of 39 convolutional layers, which includes four spatial attention modules and six channel attention modules, as shown in Figure 1. The initial learning rate is 0.01, the batch size is 16, and the number of training epochs is 30. The saved training network is the network parameter with the best accuracy on the validation dataset among the 30 training epochs.

To verify the superiority of the algorithm in this study, 2D-CNN, ResNet, and ResNet-CBAM are used for the comparison experiments. 2D-CNN is the most common network used for recognition, and ResNet is a 34-layer residual network that is used without any changes for the comparative experiments. ResNet-CBAM is used to comparatively verify the superiority of the attention mechanism designed in this study.

In detail, 2D-CNN is a two-dimensional CNN containing four convolutional layers. The size of the convolution kernel in the first layer is 13 × 13 × 64, the size of the convolution kernel in the second layer is 9 × 9 × 128, the size of the convolution kernel in the third layer is 5 × 5 × 128, and the size of the convolution kernel in the fourth layer is 3 × 3 × 256. Finally, the data are flattened, and a 1 × 6 classification result is output through the soft ax activation function. ResNet-CBAM is a 34-layer residual network with a CBAM module on each layer. To explore the recognition and classification performance of the network, the four algorithms are trained under different JNRs.

The overall accuracy (OA) and kappa coefficient (Kappa) are used to compare the abilities of the proposed algorithm. The OA and Kappa are computed as follows:

(19) $$OA=\frac{1}{N}\sum_{l=1}^{L}TP_{l}$$

where $TP_{l}$ is the number of correctly classified samples of L  class, and N is the total number of test samples.

(20) $$\mathrm{Kappa}=\frac{\sum_{i=1}^{L}a_{i}\times b_{i}}{N^{2}}$$

where the number of samples in the i class is $a_{i}$ and that predicted by the algorithm is $b_{i}$.

All experiments are carried out on a computer using Python 3.7, CUDA 10.2 version, with 32 GB RAM and an RTX4090D (Nvidia, Santa Clara, CA, USA). In addition, all the results shown are obtained by averaging the results of five independent trainings of the algorithm to eliminate the contingency of the training process and ensure the reliability of the results.

### 4.3. Results and Analysis

To validate the efficacy of the signal processing approach, specifically STFT, adopted in this study, Table 3 presents a comparison of the accuracy performances of the Mel Spectrogram, the Continuous Wavelet Transform (CWT), and the STFT. For each of these signal-processing approaches, we generated datasets under the same set of parameters to ensure a fair comparison. We utilized the identical ResNet-SCFAM network architecture for 30 epochs in 10 JNR value across all the experiments to evaluate the performance in terms of accuracy. We also compared the time that each method requires for analyzing a single identical RF signal. Specifically, for each method, the corresponding RF signal is run 100 times, and the average value of these 100 running times is taken as the value of the process time.

The results clearly demonstrated the advantages of STFT in terms of accuracy and processing time. The Mel spectrogram tended to emphasize frequency components relevant to human hearing, which did not always align well with the key features of our jamming signals. As a result, the accuracy of the recognition using the Mel method was relatively lower compared to our proposed method. For CWT, it showed good performance in capturing the time–frequency characteristics of non-stationary signals, but it still lagged behind our method in terms of overall accuracy and computational efficiency.

**Figure 4:**
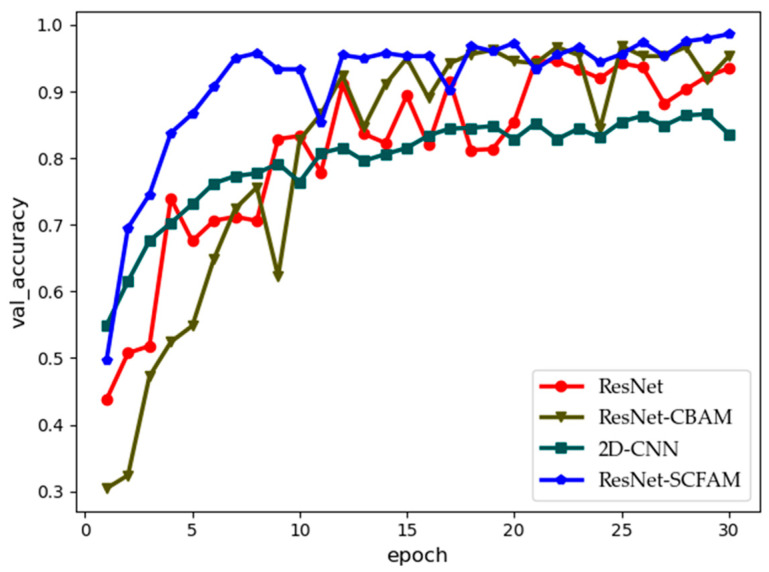
Validation accuracy of different algorithms under different epochs.

Figure 4 illustrates the performance of various algorithms across multiple epochs. When statistically comparing the recognition accuracy on the validation set over 30 training epochs, it is evident that the 2D-CNN exhibits relatively weak performance in interference recognition due to its simpler architecture, which limits its ability to extract more complex features compared to residual networks and their variants. As a classic deep residual network, ResNet-34 demonstrates moderate feature extraction capabilities. The figure shows that its performance remains at an intermediate level, with some fluctuations in performance metrics across different epochs but an overall stable trend. However, when the CBAM attention mechanism is added to ResNet-34, its accuracy in the first 10 epochs is significantly lower than that of other algorithms. This can be attributed to the increased number of parameters introduced by CBAM, which may cause unstable gradient updates and negatively impact the convergence speed of the entire network. The attention algorithm proposed in this study selectively incorporates different attention mechanisms into various layers based on the hierarchical structure of the residual network. This approach not only avoids the decrease in recognition accuracy caused by excessive parameters but also enhances both recognition accuracy and stability.

**Figure 5:**
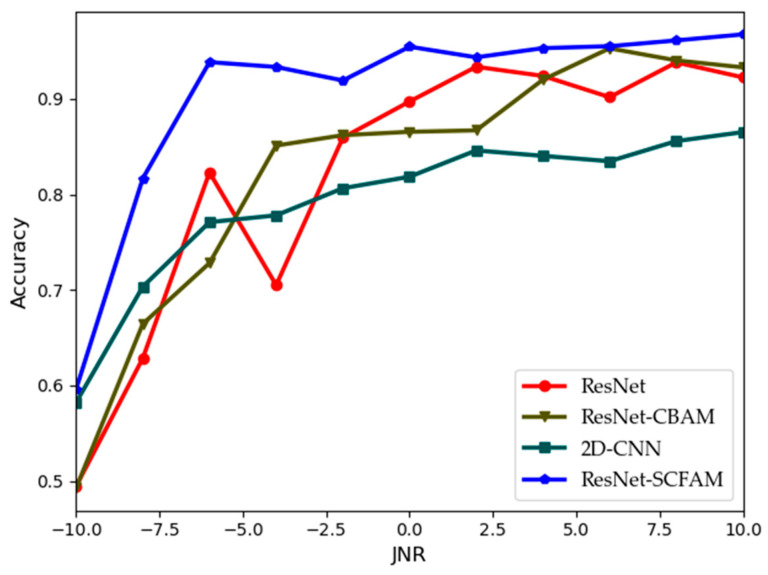
Validation accuracy of different algorithms under different JNRs.

The accuracy of four algorithms under different JNRs is shown in Figure 5. Evidently, 2D-CNN has the lowest recognition accuracy due to its simple convolution structure. ResNet, being a classic deep residual network, has the ability of extracting features. The accuracy curve of ResNet is affected to some extent due to lower JNR values. The accuracy curve of ResNet-CBAM is much lower than that of other algorithms at lower JNRs. The addition of CBAM leads to unstable gradient updates, thus affecting the accuracy. After adding the SCFAM, the performance of the proposed algorithm is significantly improved, as shown in the curve of ResNet-SCFAM. Under each JNR value, the performance of ResNet-SCFAM is better than that of ResNet. This indicates that the SCFAM module can effectively enhance the ability of an algorithm to extract and focus on features.

Furthermore, the OA and Kappa of four different algorithms are shown in Table 3. Evidently, the algorithm we proposed has a higher accuracy than other machine learning algorithms, and the proposed ResNet-SCFAM has a small standard deviation. Compared with ResNet and ResNet-CBAM, the proposed ResNet-SCFAM has an increased recognition accuracy by more than 5%, which confirms that the proposed fused attention mechanism can effectively capture more important features and improve the recognition ability.

The processing time and response time of the four algorithms are also presented in Table 4. The processing time corresponds to the total training duration over 30 epochs when utilizing the same training dataset. The response time is defined as the time elapsed from the moment the jamming signal is input into the system until the recognition result is generated during the validation phase. All experiments were conducted on the same computing platform with CUDA 11.0 to ensure consistency and fairness in the comparison. The ResNet-SCFAM algorithm demonstrates a better comprehensive performance in terms of both processing time and response time. It can not only complete task processing relatively quickly but also promptly initiate processing once the input is received. In contrast, the ResNet-CBAM algorithm shows relatively poor performance in both processing time and response time, and its computational efficiency issues need to be considered in practical applications. The 2D-CNN algorithm has the fastest response time, yet its processing time is relatively long. As for the ResNet algorithm, it exhibits a moderate performance in both aspects.

In order to study the robustness of the proposed algorithm, four new types of compound jamming are used as the test dataset. As shown in Table 5, the recognition accuracy rates of compound jamming in the test dataset are all greater than 90%. For FM + CI compound jamming, the accuracy rate can reach 97%. Nevertheless, it is impossible to recognize new types of compound jamming that have not been trained by networks for other algorithms. Thus, new types of compound jamming are only recognized as a trained compound jamming.

**Figure 6:**
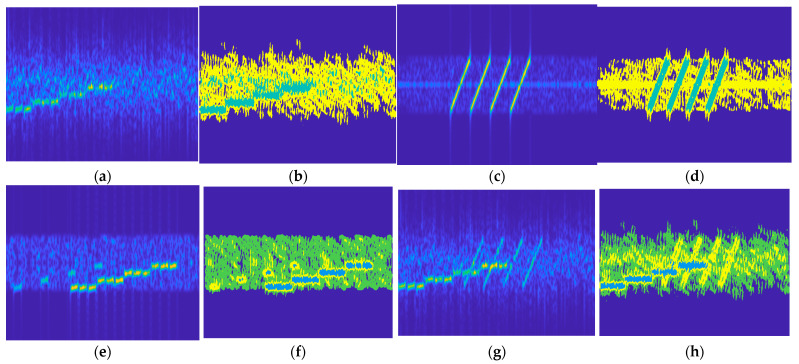
Time–frequency diagram of the segmentation results of the compound jamming in the test dataset.(**a**) FM + CI before segmentation; (**b**) FM + CI after segmentation; (**c**) AM+SMSP before segmentation; (**d**) AM + SMSP after segmentation; (**e**) RF + ISRJ + CI before segmentation; (**f**) RF + ISRJ + CI after segmentation; (**g**) FM + CI + SMSP before segmentation; (**h**) FM + CI + SMSP after segmentation.

Figure 6 presents the segmentation results of the compound jamming in the test dataset produced by the compound segmentation module. It can be clearly observed in the time–frequency diagram that the segmentation module has essentially achieved effective segmentation of diverse jamming. In particular, the segmentation performance is optimal for the various combinations of suppression jamming and deception jamming, such as FM + CI and AM + SMSP, which is consistent with the high correct recognition probability of the recognition network. It can be reasonably concluded that the greater the distinguishability of jamming features, the more significant the effect of the compound jamming recognition method proposed in this study. For three-component jamming composites like RF + ISRJ + CI and FM + CI + SMSP, since the interference mechanisms of RF and ISRJ are analogous and their statistical and texture characteristics in the time–frequency diagram are also similar, the segmentation outcome is less than satisfactory. This, in turn, results in a relatively low correct rate of the recognition network. For the composite jamming of FM + CI + SMSP, although CI and SMSP exhibit some overlap in the time–frequency spectrogram, owing to the texture disparities, the segmentation module can still achieve a relatively good segmentation effect.

## 5. Conclusions

In this study, a novel method of compound jamming recognition is proposed, which consists of the segmentation module and the ResNet-SCFAM algorithm. The proposed method has the ability to recognize a new compound jamming pattern that is untrained by a network, making it a new and effective method for jamming recognition. We compared our proposed method with other related algorithms using simulated datasets, and the results show that the proposed algorithm has a superior recognition ability in recognizing untrained types of compound jamming. In future research, a wider range of jamming signals with more complex combinations should be studied. Meanwhile, investigations into more efficient image segmentation methods are imperative. These efforts aim to boost the accuracy of compound jamming recognition, thus more comprehensively addressing jamming-related challenges in relevant fields.

## Figures and Tables

**Table 1:** Parameters of the residual network and the allocation of the attention mechanism throughout all layers.

Layer Name	Kernel Size	Stride	Padding	Activation Function	Down Sampling	Attention Mechanism
Conv1	7 × 7	2	3	RELU	/	/
maxpool	3 × 3	2	1	/	/	/
layer1-block1	3 × 3 (conv1, conv2)	1	1	RELU	/	CAM
layer1-block2	3 × 3 (conv1, conv2)	1	1	RELU	/	CAM
layer1-block3	3 × 3 (conv1, conv2)	1	1	RELU	/	CAM, SAM
layer2-block1	3 × 3 (conv1, conv2)	2	1	RELU	Yes	CAM
layer2-block2	3 × 3 (conv1, conv2)	1	1	RELU	/	/
layer2-block3	3 × 3 (conv1, conv2)	1	1	RELU	/	CAM
layer2-block4	3 × 3 (conv1, conv2)	1	1	RELU	/	/
layer3-block1	3 × 3 (conv1, conv2)	2	1	RELU	Yes	/
layer3-block2,3,4,5,6	3 × 3 (conv1, conv2)	1	1	RELU	/	/
layer4-block1	3 × 3 (conv1, conv2)	2	1	RELU	Yes	SAM
layer4-block2	3 × 3 (conv1, conv2)	1	1	RELU	/	SAM
layer4-block3	3 × 3 (conv1, conv2)	1	1	RELU	/	/
Global Pooling Layer	Adaptive averaging pooling processing					
Fully Connected Layer	Number of trainable parameters: 512 × 6					

**Table 2:** Parameter range of jamming signals.

**Dataset**	**Jamming**	**Parameters**	**Value Range**
Training and validation dataset	FM	Bandwidth	10~20 MHz
		Carrier frequency	8~12 GHz
		Frequency-modulation slope	10~20 MHz/s
		JNR	−10~10 dB
	AM	Bandwidth	10~20 MHz
		Carrier frequency	8~12 GHz
		JNR	−10~10 dB
	RF	Bandwidth	5~10 MHz
		Carrier frequency	8~12 GHz
		JNR	−10~10 dB
	ISRJ	Sampling period	4~10 us
		Sampling time	1~5 us
		JNR	−10~10 dB
	CI	Number of sub-pulses	2~8
		Number of pulse-forwarding	2~4
		JNR	−10~10 dB
	SMSP	Bandwidth	10~30 MHz
		Sampling multiple	2~4
		JNR	−10~10 dB
	FM + ISRJ	Same as corresponding jamming components	Same as corresponding jamming components
	AM + CI		
	RF + SMSP		
	ISRJ + CI		
Test dataset	FM + CI	Same as corresponding jamming components	Same as corresponding jamming components
	AM + SMSP		
	RF + ISRJ + CI		
	FM + CI + SMSP		

**Table 3:** Accuracy rate and process time of three signal processing techniques: Mel, CWT and STFT.

Signal Processing Technique	Accuracy (%)	Processing Time (s)
Mel	78.46	0.115
CWT	92.37	0.204
STFT	98.60	0.079

**Table 4:** OA, Kappa, processing time and response time of four algorithms.

Algorithms	OA (%)	Kappa (%)	Processing Time (s)	Response Time (s)
ResNet	93.18 ± 0.09	92.48 ± 0.65	599.42	0.27
ResNet-CBAM	92.27 ± 0.42	91.61 ± 0.39	825.24	0.19
2D-CNN	92.29 ± 1.39	91.09 ± 0.45	646.63	0.14
ResNet-SCFAM	98.60 ± 0.24	96.96 ± 0.60	534.82	0.15

**Table 5:** Accuracy rate of ResNet-SCFAM for compound jamming in test dataset.

Compound Jamming	ResNet-SCFAM
FM + CI	97.0%
AM + SMSP	96.5%
RF + ISRJ + CI	91.5%
FM + CI + SMSP	92.5%

## Data Availability

Data are contained within the article.
